# Combined fecal microbiota transplantation and adipose-derived stem cells protect the blood-spinal cord barrier after spinal cord injury

**DOI:** 10.3389/fimmu.2026.1877863

**Published:** 2026-10-13

**Authors:** Zhicheng Tang, Junjie Chen, Daming Peng, Biling Mo, Shun Yao, Huan Xie, Daoyuan Jiang, Wentao Zhuo, Hui Zhang, Zhilai Zhou

**Affiliations:** 1 Division of Spine, Department of Orthopedics, The Affiliated Guangdong Second Provincial General Hospital of Jinan University, Guangzhou, Guangdong, China; 2 Department of Cardiovascular, Liwan Central Hospital of Guangzhou, Guangzhou, Guangdong, China; 3 Department of Orthopedics, First Affiliated Hospital of Gannan Medical University, Ganzhou, Jiangxi, China; 4 The Second School of Clinical Medicine, Southern Medical University, Guangzhou, Guangdong, China

**Keywords:** adipose-derived stem cells, blood-spinal cord barrier, fecal microbiota transplantation, paracrine, spinal cord injury, tight junction

## Abstract

**Background:**

Blood–spinal cord barrier (BSCB) disruption and neuroinflammation contribute to secondary injury after spinal cord injury (SCI). Fecal microbiota transplantation (FMT) and human adipose-derived stem cells (hADSCs) each show therapeutic potential, but the effects of combined treatment and the influence of fecal soluble components on hADSCs secretory activity remain unclear.

**Methods:**

A T9 forceps-crush SCI model was established in C57BL/6J mice to compare FMT, intralesional hADSCs transplantation, and their combination. Functional recovery, BSCB permeability, neuroinflammation, lesion pathology, and neural tissue preservation were evaluated. Exploratory 16S rRNA sequencing was performed at 35 days post-injury. *In vitro*, hADSCs were exposed to filtered fecal material from healthy donor mice, and their conditioned medium was applied to oxygen–glucose deprivation/reoxygenation-injured bEnd.3 endothelial cells.

**Results:**

Combined FMT and hADSCs treatment improved locomotor recovery, attenuated BSCB leakage and neuroinflammatory responses, and preserved neural tissue, with several outcomes greater than either monotherapy. Exploratory 16S analysis identified treatment-associated differences in microbial richness and overall community composition. Fecal-filtrate preconditioning increased hADSCs secretion of VEGF, HGF, and IL-10 by 2.3-, 2.2-, and 1.8-fold, respectively. Conditioned medium from preconditioned hADSCs improved wound closure, preserved ZO-1 and Claudin-5 signals, and reduced endothelial monolayer permeability after OGD/R. IL-10 silencing partially attenuated these protective effects.

**Conclusions:**

Combined FMT and hADSCs treatment was associated with greater BSCB preservation, reduced neuroinflammation, and improved functional recovery after SCI. *In vitro*, soluble components of healthy-donor fecal filtrate modulated hADSCs secretory activity and enhanced endothelial protection. These findings do not establish that oral FMT reprograms transplanted hADSCs *in vivo* or that microbiota alterations mediate the combined therapeutic effect.

## 1 Introduction

Spinal cord injury (SCI) initiates a cascade of secondary damage in which blood-spinal cord barrier (BSCB) disruption plays a central role (1, 2). Within hours of mechanical trauma, spinal microvascular endothelial cells lose tight junction integrity, allowing plasma proteins, inflammatory cells, and neurotoxic factors to enter the spinal parenchyma (2, 3). This vascular breach amplifies tissue damage, promotes edema, and drives progressive neurological deterioration (1, 4, 5). Limiting BSCB disruption and preserving barrier integrity are therefore considered important therapeutic objectives in SCI research (2, 6, 7).

Human Adipose-derived stem cells (hADSCs) have attracted interest as a cell-based therapy for SCI because of their accessibility, safety profile, and paracrine activity (8–10). Transplanted hADSCs secrete multiple trophic and immunomodulatory factors, including vascular endothelial growth factor (VEGF), hepatocyte growth factor (HGF), interleukin-10 (IL-10), and tumor necrosis factor-stimulated gene-6 (TSG-6), which have been implicated in the regulation of inflammation, angiogenesis, and neural tissue preservation (9, 11–13). Despite these properties, the therapeutic efficacy of hADSCs transplantation alone remains variable and is often incomplete (8). One potential limitation is that the injured spinal cord creates a hostile microenvironment that may compromise hADSCs persistence and alter their paracrine activity (14–16).

Previous studies have shown that SCI is accompanied by alterations in the gut microbiota, which have been associated with altered metabolite profiles and systemic inflammatory responses that may influence neurological outcomes (17–20). SCI-associated gut dysbiosis has also been associated with systemic complications beyond the central nervous system, highlighting the broader consequences of intestinal microbial alterations after SCI (21). Fecal microbiota transplantation (FMT) has shown therapeutic potential after SCI and has been associated with changes in the gut microbial environment and the spinal cord microenvironment (20, 22, 23). These effects may involve alterations in microbial communities and microbiota-derived metabolites, including short-chain fatty acids, although the pathways linking intestinal microbial changes to spinal cord repair remain incompletely understood (19, 20, 23). Although FMT and hADSCs transplantation have each demonstrated therapeutic potential after SCI, whether their combination provides greater therapeutic benefit than either intervention alone has not been established.

Combining FMT with hADSCs transplantation may provide complementary therapeutic effects by targeting different components of secondary injury after SCI. One potential link between these interventions is the ability of mesenchymal stem cells to respond to extracellular metabolic and inflammatory cues. Previous studies have shown that microbial metabolites and other preconditioning stimuli can modulate mesenchymal stem cell secretory activity (24–26). This raises the possibility that soluble components associated with the intestinal microbial environment may influence hADSCs paracrine function. However, whether such an interaction occurs after FMT *in vivo* remains unknown.

In the present study, we evaluated whether combining FMT with intralesional hADSCs transplantation provides greater therapeutic benefit than either treatment alone in a mouse forceps-crush SCI model. We assessed functional recovery, BSCB disruption, neuroinflammation, chronic lesion pathology, and neural tissue preservation. Exploratory 16S rRNA sequencing was used to characterize treatment-associated differences in gut microbial composition among four SCI groups. In parallel, we used a reductionist *in vitro* model to determine whether filtered fecal material from healthy donor mice could alter hADSCs secretory activity and whether conditioned medium from fecal-filtrate-preconditioned hADSCs provided greater protection to oxygen–glucose deprivation/reoxygenation (OGD/R)-injured endothelial cells. This *in vitro* system was used to examine the responsiveness of hADSCs to soluble components of healthy-donor fecal material and was considered complementary to, rather than a direct model of, the *in vivo* interaction between oral FMT and transplanted hADSCs.

## 2 Materials and methods

### 2.1 Study design

This study combined an *in vivo* SCI experiment with complementary *in vitro* assays. The *in vivo* study compared FMT, hADSCs transplantation, and their combination with respect to functional recovery, BSCB disruption, neuroinflammation, chronic tissue pathology, and neural tissue preservation. Exploratory 16S rRNA sequencing was performed to characterize treatment-associated differences in gut microbial composition among four SCI groups. In parallel, a reductionist *in vitro* model was used to examine whether filtered fecal material from healthy donor mice altered hADSCs secretory activity and whether conditioned medium from preconditioned hADSCs protected OGD/R-injured endothelial cells. IL-10 knockdown was further used to examine the contribution of hADSCs-derived IL-10 to these *in vitro* effects.

### 2.2 hADSCs isolation, culture, and characterization

Human adipose-derived stem cells (hADSCs) were isolated from lipoaspirates obtained from Guangdong Second Provincial General Hospital under approved ethical protocols with informed donor consent (Approval No. 2025-KY-KZ-277-02). Cells were prepared by enzymatic digestion and differential centrifugation, as previously described, and maintained at 37 °C in a humidified atmosphere with 5% CO_2_ using high-glucose DMEM supplemented with 10% fetal bovine serum and 1% antibiotic–antimycotic reagent (27). Cells at passages 3–5 were used.

Mesenchymal identity was verified by flow cytometry and trilineage differentiation. hADSCs were positive for CD73, CD105, and CD90 and negative for CD14, CD45, CD34, CD19, and HLA-DR. The chondrogenic, adipogenic, and osteogenic differentiation capacities were assessed using commercial induction kits (PD-007, PD-006, and PD-020; Pricella), followed by Alcian Blue, Oil Red O, and Alizarin Red S staining, respectively.

### 2.3 Animals and experimental groups

All experimental protocols involving animals were approved by the Institutional Animal Care and Use Committee of Guangdong Second Provincial General Hospital (Approval No. 2025-DW-KZ-023-01). Specific-pathogen-free female C57BL/6J mice (6–8 weeks old, 20 ± 2 g) were obtained from BesTest Bio-Tech Co., Ltd. (Zhuhai, China; License No. SCXK 2025-0051). Animals were maintained under a 12-h light/dark cycle with free access to standard chow and water.

In total, 130 mice were allocated using a computer-generated randomization sequence to the study groups specified for each experimental endpoint. Nineteen animals died before their scheduled endpoints, leaving 111 animals available for the prespecified endpoint analyses. The order of surgery, behavioral testing, and tissue processing was balanced across groups, and cage positions were rotated regularly. Behavioral, histological, and molecular evaluations were performed by investigators blinded to group assignment. No formal prospective power calculation was performed; group sizes were based on previous SCI studies using comparable outcome measures and were predetermined before data collection. Animal enrollment, mortality, endpoint allocation, and final sample sizes are summarized in **Supplementary Figure S1**.

### 2.4 SCI induction and treatments

#### 2.4.1 Forceps-crush SCI model

SCI was induced as previously described (28). Mice were anesthetized with 2–3% isoflurane, and a laminectomy was performed at T9. The exposed spinal cord was bilaterally crushed for 2 s using fine forceps (0.1-mm tips; Dumont #5). Sham mice underwent laminectomy without compression.

Immediately after surgery, all animals received a single subcutaneous dose of ampicillin (30 mg/kg in 100 μL saline). No additional postoperative antibiotics were administered. Manual bladder expression until spontaneous voiding recovered. At 7 or 35 d.p.i., the mice were transcardially perfused first with saline and subsequently with 4% paraformaldehyde. A spinal cord segment containing the lesion epicenter was collected for subsequent analyses.

#### 2.4.2 hADSCs transplantation

hADSCs transplantation was performed immediately after SCI induction, as previously described (27). Mice in the SCI + hADSCs and SCI + FMT + hADSCs groups received an intralesional injection of 1 × 10^6^ hADSCs suspended in 2 μL PBS at the lesion epicenter. Mice in the SCI and SCI + FMT groups received an equivalent volume of PBS at the same site as the vehicle control. No pharmacological immunosuppression was administered before or after hADSCs transplantation.

#### 2.4.3 Fecal microbiota transplantation

FMT was performed as previously described (20). Fresh fecal pellets from healthy donor mice were homogenized in sterile saline to obtain a final concentration of 10% (w/v), centrifuged, and the supernatant was used as the FMT suspension. Mice in the SCI + FMT and SCI + FMT + hADSCs groups received 100 μL of freshly prepared supernatant by oral gavage once daily, beginning on the day of injury and continuing until sacrifice at 7 or 35 d.p.i. Mice in the Sham, SCI, and SCI + hADSCs groups received an equal volume of sterile saline on the same schedule.

### 2.5 Behavioral assessments

Motor and sensory recovery after SCI were evaluated using the Basso Mouse Scale (BMS), footprint analysis, von Frey testing, hot-plate testing, and the inclined plane test. All behavioral assessments were independently conducted by three trained investigators who were unaware of the group assignments.

#### 2.5.1 Basso mouse scale

Hindlimb motor performance was evaluated with the 9-point BMS, in which scores of 0 and 9 represent complete paralysis and normal locomotion, respectively. Assessments were performed before surgery and at 1, 3, 5, 7, 14, 21, 28, and 35 days after surgery during a 4-min open-field test following a 5-min acclimation period.

#### 2.5.2 Footprint analysis

At 35 d.p.i., gait and interlimb coordination were evaluated using footprint recording. Forepaws and hindpaws were coated with different colored non-toxic dyes, and mice were allowed to traverse a paper-lined runway.

#### 2.5.3 von Frey test

Mechanical withdrawal thresholds were assessed using calibrated von Frey filaments applied to the plantar surface of the hind paw. The 50% paw withdrawal threshold was measured before surgery and at 1, 3, 5, 7, 14, 21, 28, and 35 days postoperatively.

#### 2.5.4 Hot-plate test

Thermal pain sensitivity was tested with a hot plate set at 52.0 ± 0.5 °C. The latency to paw withdrawal was measured, with a 30-s cutoff imposed to prevent tissue damage. Assessments were carried out before surgery and at postoperative days 1, 3, 5, 7, 14, 21, 28, and 35.

#### 2.5.5 Inclined plane test

At 35 d.p.i., trunk and limb strength were evaluated using the inclined plane test by measuring the maximum angle at which each mouse was able to remain in position for 5-s.

### 2.6 Histology and immunofluorescence

For histological assessment, mice were deeply anesthetized at 7 or 35 days post-injury and subjected to transcardial perfusion with PBS, followed by fixation using 4% paraformaldehyde. A 1-cm spinal cord section centered on the lesion epicenter was harvested, post-fixed, cryoprotected in graded sucrose solutions, and serially sectioned in the sagittal plane at a thickness of 12 μm (27). Approximately 15 sagittal sections spanning the lesion-centered region were collected from each animal. For each staining modality, three sections per animal were selected at approximately 40-μm intervals to provide spatially separated sampling across the lesion.

#### 2.6.1 Histological staining

Sections were processed with commercially available staining kits (Servicebio) following the protocols provided by the manufacturer: hematoxylin and eosin (H&E; G1005) for general histopathology, Nissl staining (G1086) for neuronal morphology, Masson’s trichrome staining (G1006) for collagen deposition and fibrotic scar assessment, and Luxol fast blue with Cresyl Violet counterstaining (G1030) for myelin preservation. Quantitative measurements from the three sections were averaged to generate a single biological value for each animal. The collagen-positive area in Masson-stained sections was quantified using ImageJ and used as a measure of fibrotic scar area.

#### 2.6.2 Immunofluorescence staining

Sagittal spinal cord cryosections were selected according to the section-sampling procedure described above. Sections were fixed, permeabilized with Triton X-100, and blocked with 10% donkey serum. They were then incubated overnight at 4 °C with the primary antibodies listed in **Table 1**, including antibodies against CD68, Iba1, TNF-α, IL-1β, NF200, NeuN, ZO-1, Claudin-5, CD31, GFAP, and human nuclear antigen (HuNu), as appropriate for each experiment. After washing, sections were incubated with species-appropriate Alexa Fluor-conjugated secondary antibodies (Invitrogen; 1:300) for 2 h at room temperature in the dark. Nuclei were counterstained with DAPI. Confocal images were acquired using a Leica Stellaris 5 microscope under identical acquisition settings for all groups within each experiment.

**Table 1:** Primary antibodies for immunofluorescence, with applied dilutions.

No.	Antibody	Working dilution	Manufacturer	Catalog number
1	anti-NeuN	1:200	Sigma	MAB377
2	anti-NF200	1:400	Abcam	ab8135
3	anti-IL-1β	1:200	ABclonal	A22257
4	anti-TNF-α	1:100	Santa Cruz	sc-52746
5	anti-CD68	1:200	Bio-Rad	MCA1957GA
6	anti-CD31/PECAM-1	1:300	BD Biosciences	550274
7	anti-ZO-1	1:200	ABclonal	A25306
8	anti-Claudin-5	1:200	ABclonal	A25830
9	anti-GFAP	1:800	Sigma	mab360
10	anti-Iba-1	1:200/1:500	Abcam/ Wako	ab283319/ 019–19741
11	anti-HuNu	1:100	Millipore	MAB1281

#### 2.6.3 Immunofluorescence quantification

Quantitative immunofluorescence analyses were performed using Leica LAS X software and ImageJ. For each staining modality, measurements obtained from the selected sections and fields were averaged to generate a single biological value for each animal. For acute inflammatory markers, CD68-, TNF-α-, and IL-1β-positive areas were quantified within predefined lesion-centered regions of interest and expressed as a percentage of the analyzed tissue area. For double-immunofluorescence analysis, Iba1-positive regions were first segmented, and the TNF-α- or IL-1β-positive area contained within the Iba1-positive regions was quantified and expressed as a percentage of the corresponding Iba1-positive area.

For BSCB-associated junctional proteins, CD31-positive microvessels were used to define the vascular mask. The areas of ZO-1 or Claudin-5 signal overlapping with the CD31-positive vascular area were quantified and expressed as the percentage of CD31-positive vascular area showing ZO-1 or Claudin-5 overlap. NF200 immunofluorescence intensity was quantified after background subtraction within predefined perilesional regions and expressed relative to the Sham group. NeuN-positive cells were manually counted within predefined regions at specified distances from the lesion center. At 35 d.p.i., CD68 fluorescence intensity in GFAP/CD68-stained sections was quantified after background subtraction and expressed relative to the Sham group. HuNu-positive cells within and around the lesion were quantified in the SCI + hADSC and SCI + FMT + hADSC groups (n = 6 animals per group) and expressed as cells/mm². All image acquisition and quantitative analyses were performed using identical settings within each staining experiment.

### 2.7 Evans blue assay

BSCB permeability was assessed at 7 d.p.i. using Evans blue (EB) extravasation (29). Mice received an intravenous injection of 2% EB solution (0.2 mL; Sigma-Aldrich), and the dye was permitted to circulate for 60 min. Mice were then perfused transcardially with saline to clear intravascular dye. Spinal cords were harvested, photographed under standardized illumination, and imaged with an IVIS Spectrum system (PerkinElmer) for near-infrared fluorescence quantification (ex/em 620/680 nm).

### 2.8 Transwell-based FITC–dextran permeability assay

The barrier function of bEnd.3 endothelial monolayers was assessed by measuring the transendothelial passage of 70-kDa fluorescein isothiocyanate–dextran (FITC–dextran; Sigma-Aldrich), as previously described (6). bEnd.3 cells were seeded onto Transwell inserts (Millipore) and cultured until a confluent monolayer was established. Following the indicated OGD/R and conditioned-medium treatments, FITC–dextran was added to the apical compartment at a final concentration of 1 mg/mL. After incubation at 37 °C for 60 min, 100 μL of medium was collected from the basolateral compartment, and fluorescence was measured using a BioTek fluorescence microplate reader at excitation/emission wavelengths of 485/525 nm. Barrier permeability was quantified according to the fluorescence intensity of FITC–dextran detected in the basolateral compartment and expressed relative to the corresponding control condition.

### 2.9 qPCR and ELISA

#### 2.9.1 qPCR

Total RNA was isolated from spinal cord samples using a commercial RNA extraction kit (M5101, NCM Biotech) following the manufacturer’s instructions. RNA concentration and purity were determined spectrophotometrically. Equal amounts of RNA were subjected to reverse transcription to generate cDNA using the GenStar cDNA Synthesis Kit (A232-10). Quantitative real-time PCR was then performed using SYBR Green Master Mix (A301-01, GenStar). Relative mRNA expression was normalized against GAPDH and calculated using the 2^−ΔΔCt^ method. Primer sequences are listed in **Table 2**.

**Table 2:** Sequences of primer pairs.

Genes	Forward primer sequence (5′– 3′)	Reverse primer sequence (5′– 3′)
IL-18	5′-ATGCCTGATATCGACCGAAC-3′	5′-TGGCACACGTTTCTGAAAGA-3′
IL-1β	5′-CAGCAGCATCTCGACAAGAG-3′	5′-CATCATCCCACGAGTCACAG-3′
TNF-α	5′-TACTGAACTTCGGGGTGATCG-3′	5′-CCACTTGGTGGTTTGCTACG-3′
IL-6	5′-CTTCCATCCAGTTGCCTTCTTG-3′	5′-AATTAAGCCTCCGACTTGTGAAG-3′
IL-10	5′-CACTCCCTGCATTACAATC-3′	5′-CAATGATGGTATTATAGGATCCC-3′
GAPDH	5′-GAACATCATCCCTGCATCCA-3′	5′-CCAGTGAGCTTCCCGTTCA-3′

#### 2.9.2 ELISA

Spinal cord homogenates collected at 7 d.p.i. were assayed for IL-18, IL-1β, IL-6, IL-10, and TNF-α with commercially available ELISA kits (Servicebio) in accordance with the manufacturer’s protocol. Absorbance at 450 nm was measured using a BioTek Synergy H1 microplate reader, and cytokine levels were derived from the corresponding standard curves.

### 2.10 Exploratory gut microbiota profiling

Fecal samples were collected at 35 days post-injury from the SCI, SCI+FMT, SCI+hADSCs, and SCI+FMT+hADSCs groups (n=9 per group). All 36 samples were collected at the same experimental endpoint and processed together in a single batch for DNA extraction, library preparation, and sequencing. Sham, pre-injury baseline, and donor fecal microbiota samples were not available for sequencing; therefore, this analysis was considered an exploratory cross-sectional comparison among the four SCI groups.

Microbial DNA was extracted using the MagPure Stool DNA KF Kit B (Magen, China). The V3–V4 region of the bacterial 16S rRNA gene was amplified using primers 338F (5′-ACTCCTACGGGAGGCAGCAG-3′) and 806R (5′-GGACTACHVGGGTWTCTAAT-3′). The resulting amplicons were subjected to 300-bp paired-end sequencing (PE300) on the MGISEQ-2000 platform by BGI Genomics (Shenzhen, China). Following quality filtering, read merging, and chimera removal, high-quality sequences were clustered into operational taxonomic units at 97% similarity using USEARCH (V7.0.1090). Taxonomy was assigned using the RDP classifier against the SILVA database (V138) with a confidence threshold of 0.6.

ACE and Chao1 richness were calculated from the normalized OTU table. Group differences were assessed using the Kruskal–Wallis test followed by Dunn’s test with Benjamini–Hochberg correction. Bray–Curtis principal coordinate analysis was used to evaluate community composition. Overall and pairwise differences were assessed using PERMANOVA with 9,999 permutations, with Benjamini–Hochberg correction for pairwise comparisons. Homogeneity of multivariate dispersion was evaluated using PERMDISP.

Phylum- and genus-level relative abundance profiles are presented as individual-sample stacked bar plots. The ten taxa with the highest overall mean abundance are shown, and the remaining taxa are grouped as “Other taxa.” These composition profiles were interpreted descriptively.

### 2.11 *In vitro* assays

#### 2.11.1 Preparation of filtered donor fecal material

Bacteria-free fecal filtrate was prepared as described (30). Briefly, freshly collected fecal samples (100 mg) from healthy donor mice were homogenized in 600 μL of sterile PBS by vortexing for 30 s, followed by centrifugation at 3000 × g for 10 min. The resulting supernatant was passed through a 0.22-μm membrane, aliquoted, and stored at −80 °C until use.

#### 2.11.2 hADSCs treatment and conditioned medium collection

Passage 3–5 hADSCs were seeded in six-well plates at approximately 2 × 10^5^ cells per well to achieve 50–60% confluence. Cells were assigned to the indicated treatment conditions, including PBS vehicle control, filtered fecal material, and IL-10 knockdown followed by filtered fecal material exposure. IL-10 knockdown was performed as previously described, with minor modifications (31). hADSCs were transfected with 40 pmol per well of human IL-10-targeting siRNA (sc-39634; Santa Cruz Biotechnology) using 4 μL per well of siRNA Transfection Reagent (sc-29528; Santa Cruz Biotechnology). The siRNA and transfection reagent were separately diluted in siRNA transfection medium, combined, and incubated for 20 min at room temperature before being applied to the cells. After 6 h, an equal volume of complete medium was added, and the cells were cultured for an additional 24 h before exposure to 5% (v/v) fecal filtrate.

After the preconditioning period, the filtrate-containing medium was completely removed. Cells were washed three times with sterile PBS and then cultured in fresh medium for 48 h. PBS-treated control cells underwent the same washout and medium-replacement procedure. Conditioned medium (CM) was subsequently collected, centrifuged at 1,000 × g for 10 min, aliquoted, and stored at −80 °C until use. IL-10 knockdown was verified by measuring human IL-10 concentrations in the CM by ELISA. Cellular metabolic activity was assessed using a CCK-8 assay kit (Dojindo).

#### 2.11.3 ELISA for paracrine factors

Levels of VEGF, HGF, IL-10, BDNF, and TSG-6 in the conditioned medium collected from ADSCs were determined with ELISA kits (R&D Systems) according to the manufacturer’s instructions. The assay procedure, including incubation steps and washing, followed the same protocol described in section 2.9.2. Absorbance values were recorded at 450 nm, and concentrations were calculated from the corresponding standard curves and expressed as pg/mL of conditioned medium.

#### 2.11.4 OGD/R injury and CM treatment of bEnd.3 cells

Mouse brain microvascular endothelial bEnd.3 cells were maintained in DMEM supplemented with 10% FBS and 1% antibiotic–antimycotic reagent. For the OGD/R model, confluent cell monolayers were incubated in glucose-free DMEM under hypoxic conditions (95% N_2_ and 5% CO_2_) for 4 h, followed by reoxygenation in high-glucose DMEM containing 10% FBS under normoxic conditions for 24 h. During reoxygenation, bEnd.3 cells received one of the following treatments: regular medium (OGD/R control), conditioned medium from untreated hADSCs, conditioned medium from fecal-filtrate-preconditioned hADSCs, or conditioned medium from IL-10-silenced hADSCs subsequently preconditioned with fecal filtrate. Conditioned media were diluted 1:1 with culture medium before application. Normoxic cells maintained under standard culture conditions served as the control group.

#### 2.11.5 Immunofluorescence staining

Following conditioned medium treatment, bEnd.3 cells were fixed in 4% paraformaldehyde for 15 min, permeabilized using 0.1% Triton X-100, and then blocked with 5% donkey serum. The following experimental steps were conducted in accordance with the protocol detailed in Section 2.6.2. Fluorescence intensity was analyzed with ImageJ.

#### 2.11.6 Wound healing assay

bEnd.3 cells were seeded in 6-well plates and grown to confluence. A scratch wound was created with a sterile 200-μL pipette tip (32). Cells were subjected to OGD/R and CM treatments as described above. Wound images were captured at 0 h and 12 h. Wound closure area was measured with ImageJ and expressed as percentage reduction from initial wound area. This assay was used to evaluate overall wound closure and was not considered a migration-specific assay.

### 2.12 Statistical analysis

Statistical analyses were performed using GraphPad Prism 9.0 and R software, as appropriate. Data are presented as mean ± standard deviation (SD). Longitudinal behavioral data were analyzed using two-way repeated-measures ANOVA followed by Bonferroni-adjusted multiple comparisons. Multiple-group comparisons at a single time point were analyzed using one-way ANOVA followed by Bonferroni’s multiple-comparison test, and two-group comparisons were performed using an unpaired two-tailed Student’s t-test. NeuN-positive cell counts according to distance from the lesion center were analyzed using two-way ANOVA with Bonferroni-adjusted comparisons.

Parametric tests were applied to histological, immunofluorescence, and molecular datasets without formal testing of normality or homogeneity of variance. For analyses with n = 3 per group (**Figures 1E**; **2D–F**), distributional assumptions could not be reliably assessed; these results were therefore interpreted cautiously because of the small sample size. Bonferroni correction was applied to *post hoc* pairwise comparisons within each outcome, whereas no additional multiplicity correction was applied across different outcome measures.

**Figure 1:**
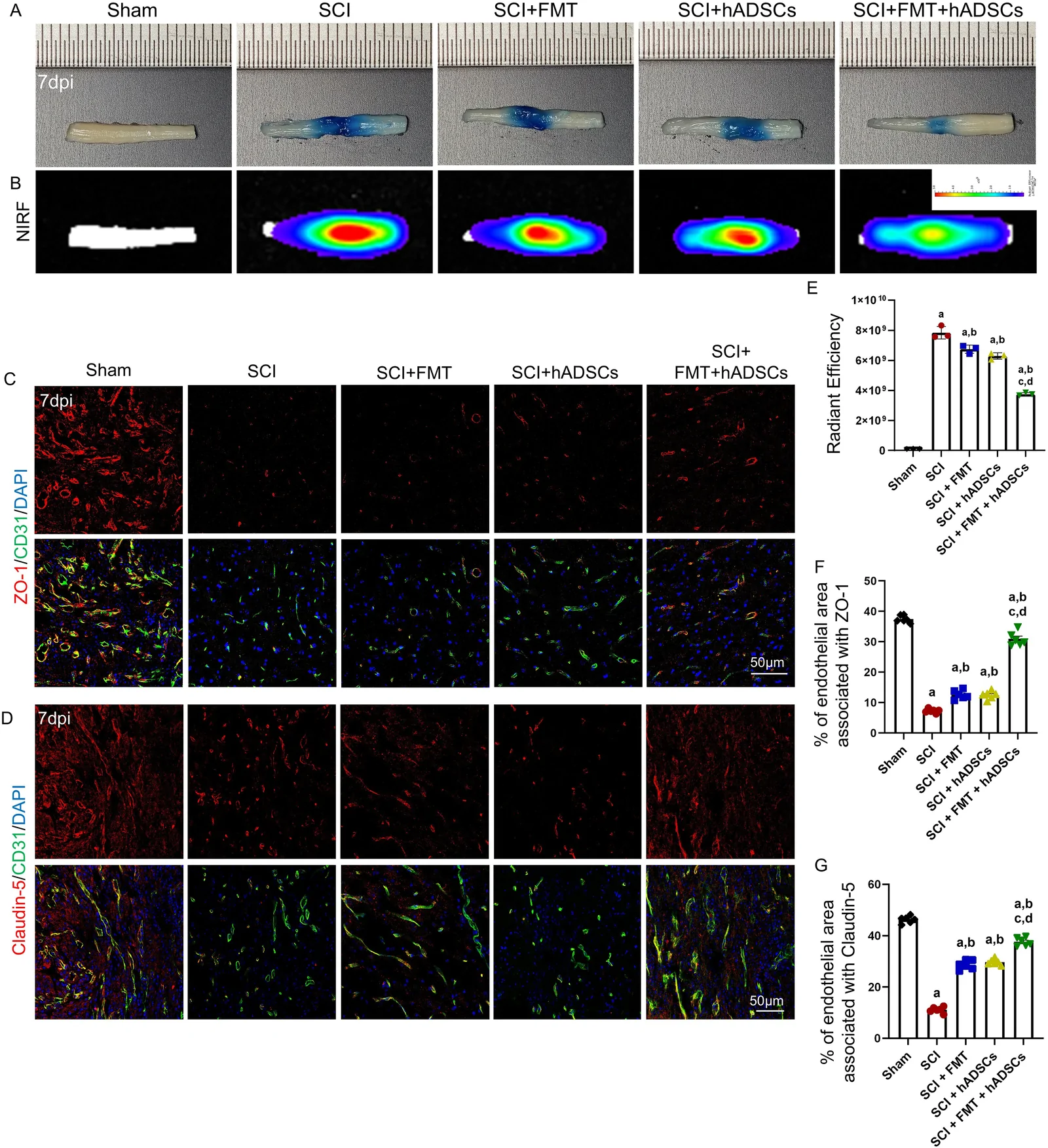
Combined FMT and hADSCs treatment attenuated acute BSCB disruption after SCI. **(A)** Representative gross images of Evans blue extravasation in spinal cord specimens at 7 d.p.i. **(B)** Near-infrared fluorescence (NIRF) imaging of the same specimens. **(C)** Representative immunofluorescence images of ZO-1 (red) and CD31 (green) in the injured spinal cord. **(D)** Representative immunofluorescence images of Claudin-5 (red) and CD31 (green). **(E)** Quantification of NIRF radiant efficiency. n = 3 animals per group. **(F)** Quantification of the percentage of CD31-positive vascular area overlapping with ZO-1 (n = 6 animals per group). **(G)** Quantification of the percentage of CD31-positive vascular area overlapping with Claudin-5 (n = 6 animals per group). Data are presented as mean ± SD. Statistical comparisons were performed using one-way ANOVA followed by Bonferroni’s multiple-comparison test. a, *P* < 0.05 vs. Sham; b, *P* < 0.05 vs. SCI; c, *P* < 0.05 vs. SCI + FMT; d, *P* < 0.05 vs. SCI + hADSCs. Scale bars, 50 μm.

**Figure 2:**
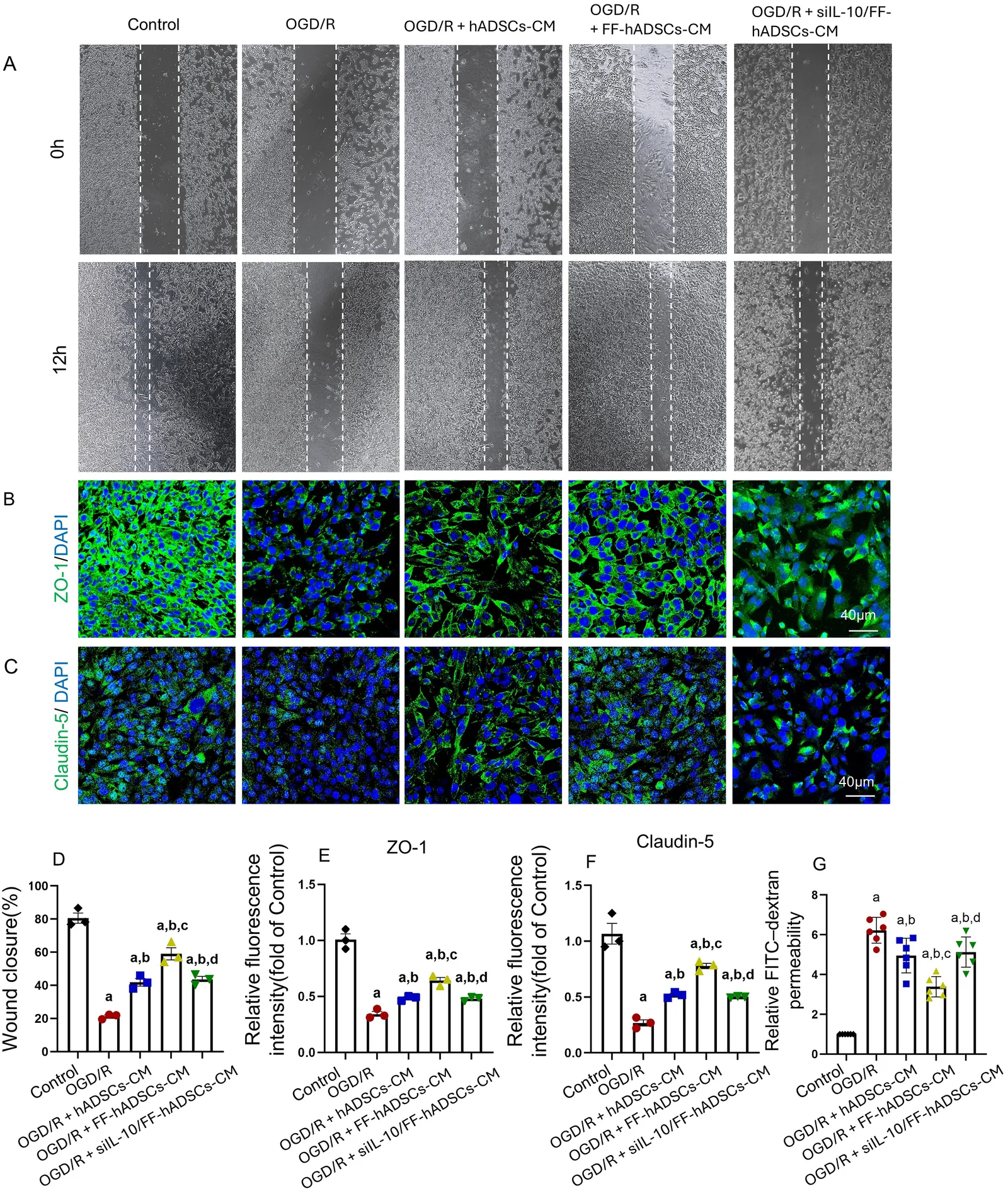
Fecal-filtrate-preconditioned hADSCs-CM enhanced endothelial protection after OGD/R injury, and IL-10 silencing partially attenuated these effects. **(A)** Representative images of wound closure at 0 and 12 h. **(B, C)** Representative immunofluorescence images of ZO-1 **(B)** and Claudin-5 **(C)**. **(D)** Quantification of wound closure, expressed as a percentage of the initial wound area. **(E, F)** Quantification of relative ZO-1 **(E)** and Claudin-5 **(F)** fluorescence intensity, normalized to the Control group. **(G)** Quantification of FITC–dextran permeability across bEnd.3 monolayers. Data are presented as mean ± SD. n = 3 independent experiments per group for panels **(D-F)** and n = 6 independent experiments per group for panel **(G)** Statistical comparisons were performed using one-way ANOVA followed by Bonferroni’s multiple-comparison test. a, *P* < 0.05 vs. Control; b, *P* < 0.05 vs. OGD/R; c, *P* < 0.05 vs. OGD/R + hADSCs-CM; d, *P* < 0.05 vs. OGD/R + FF-hADSCs-CM. FF-hADSCs-CM, conditioned medium collected from hADSCs preconditioned with filtered donor fecal material.

For the exploratory microbiota analysis, ACE and Chao1 richness indices were compared using the Kruskal–Wallis test followed by Dunn’s pairwise comparisons with Benjamini–Hochberg correction. Bray–Curtis dissimilarities were visualized by principal coordinate analysis (PCoA), and overall and pairwise differences in community composition were assessed using PERMANOVA with 9,999 permutations, with Benjamini–Hochberg correction for pairwise comparisons. Homogeneity of multivariate dispersion was evaluated using PERMDISP. For animal experiments, the individual animal was considered the biological unit of analysis, and measurements from multiple sections or fields were averaged to generate one value per animal. For *in vitro* experiments, n represents independent experiments, with technical replicate wells averaged within each experiment. P < 0.05 was considered statistically significant.

## 3 Results

### 3.1 Combined FMT and hADSCs treatment improved functional outcomes after SCI

hADSCs were characterized before transplantation (**Supplementary Figure S2**). Combined FMT and hADSC treatment showed the greatest improvement in functional outcomes among the treatment groups. At 1 d.p.i., BMS scores were comparable among the four SCI groups (all *P* > 0.05), indicating similar initial injury severity. Footprint analysis at 35 d.p.i. showed that the SCI + FMT + hADSCs group displayed longer stride length and more coordinated gait than the SCI, SCI + FMT, or SCI + hADSCs groups (**Figure 3A**). BMS scores showed a similar pattern: from 14 d.p.i. onward, the combined-treatment group scored significantly higher than either monotherapy group (**Figure 3B**). In the hot-plate and von Frey tests, the combined-treatment group also showed greater improvement in thermal withdrawal latency and mechanical withdrawal threshold than the other SCI groups (**Figures 3C, D**). In the inclined plane test at 35 d.p.i., the combined-treatment group reached 38.98 ± 0.91°, compared with 27.91 ± 1.64° in the SCI group, and also performed significantly better than either monotherapy group (**Figure 3E**).

**Figure 3:**
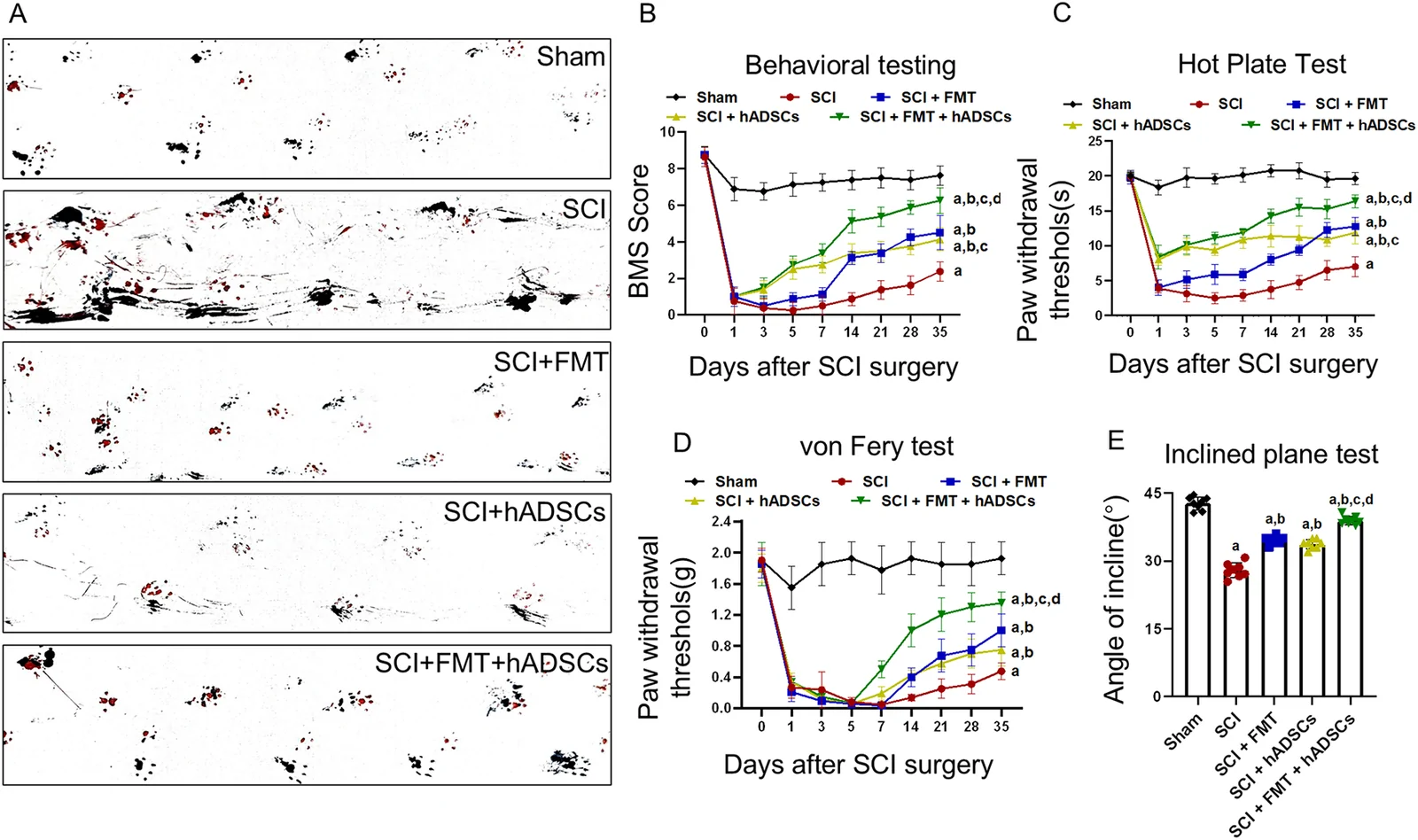
Combined FMT and hADSC treatment improved functional recovery after SCI. **(A)** Representative footprint recordings at 35 d.p.i. Forepaws and hindpaws are shown in red and black, respectively. **(B)** Basso Mouse Scale (BMS) scores over 35 days after SCI. **(C)** Thermal withdrawal latency assessed by hot-plate test. **(D)** Mechanical withdrawal threshold assessed by von Frey test. **(E)** Inclined plane test at 35 d.p.i. Data are shown as mean ± SD. n = 8 animals per group. Two-way repeated-measures ANOVA with Bonferroni correction **(B–D)**. One-way ANOVA with Bonferroni correction **(E)**. ^a^P < 0.05 vs. Sham; ^b^P < 0.05 vs. SCI; ^c^P < 0.05 vs. SCI + FMT; ^d^P < 0.05 vs. SCI + hADSCs.

### 3.2 Combined FMT and hADSCs treatment attenuated acute BSCB disruption after SCI

BSCB integrity was assessed at 7 d.p.i. by Evans blue extravasation and tight junction protein immunostaining. Gross examination and NIRF imaging showed marked Evans blue leakage in the SCI group, which was reduced by both monotherapies and most strongly by the combined treatment (**Figures 1A, B**). Radiant efficiency in the SCI group reached (7.84 ± 0.42) × 10^9^, compared with (3.75 ± 0.10) × 10^9^ in the combined-treatment group (**Figure 1E**). ZO-1 and Claudin-5 staining was continuous along CD31^+^ vessels in Sham mice but was markedly disrupted after SCI (**Figures 1C, D**). CD31-based area analysis showed that the percentage of CD31-positive vascular area overlapping with ZO-1 decreased from 37.47 ± 1.18% in the Sham group to 7.26 ± 0.64% after SCI. FMT and hADSCs monotherapies increased this proportion to 12.58 ± 1.46% and 12.41 ± 1.32%, respectively, whereas combined treatment increased it to 30.93 ± 2.26%. Similarly, the percentage of CD31-positive vascular area overlapping with Claudin-5 decreased from 46.24 ± 1.38% in Sham mice to 11.11 ± 1.13% after SCI and increased to 28.60 ± 1.77%, 29.75 ± 1.19%, and 37.64 ± 1.59% following FMT, hADSCs, and combined treatment, respectively. The combined-treatment group showed significantly greater endothelial-associated ZO-1 and Claudin-5 coverage than the SCI and both monotherapy groups (all P < 0.05; **Figures 1F, G**).

### 3.3 Combined FMT and hADSCs treatment reduced acute neuroinflammation after SCI

At 7 d.p.i., CD68, TNF-α, and IL-1β immunoreactivity was markedly increased in the SCI group (**Figures 4A–F**). The CD68-positive area was 12.53 ± 2.43% in SCI mice, compared with 2.47 ± 0.60% in the combined-treatment group. Similar reductions were observed for TNF-α (SCI: 20.24 ± 1.74%; combination: 4.90 ± 0.67%) and IL-1β (SCI: 29.78 ± 3.39%; combination: 5.40 ± 0.63%). Both monotherapy groups showed intermediate values, with reductions of approximately 50–60% relative to the SCI group (all P < 0.05).

**Figure 4:**
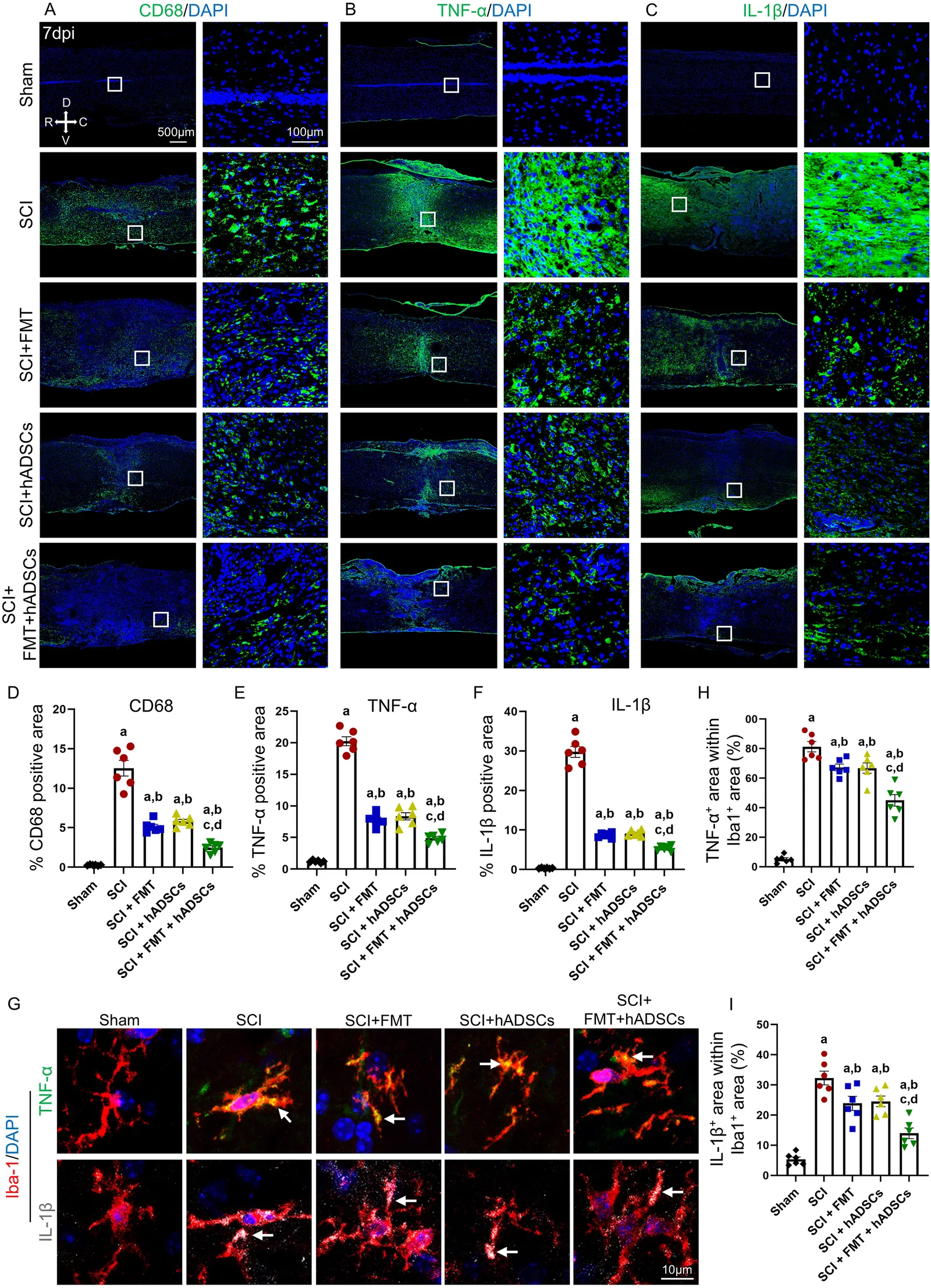
Combined FMT and hADSCs treatment reduced acute neuroinflammation after SCI. **(A–C)** Representative immunofluorescence images of CD68 **(A)**, TNF-α **(B)**, and IL-1β **(C)** in spinal cord sections at 7 d.p.i. Left panels show low-magnification views, and right panels show higher-magnification images of the boxed regions. **(D–F)** Quantification of CD68-positive area **(D)**, TNF-α-positive area **(E)**, and IL-1β-positive area **(F)**. **(G)** Representative double-immunofluorescence images showing Iba-1 (red) with TNF-α (green; upper panels) or IL-1β (white; lower panels) in spinal cord sections at 7 d.p.i. DAPI (blue) was used for nuclear counterstaining. White arrows indicate regions of overlapping Iba1 and cytokine signals. **(H)** Quantification of TNF-α-positive area within Iba1-positive regions. **(I)** Quantification of IL-1β-positive area within Iba1-positive regions. n = 6 animals per group. Data are presented as mean ± SD. Statistical comparisons were performed using one-way ANOVA followed by Bonferroni’s multiple-comparison test. a, *P* < 0.05 vs. Sham; b, *P* < 0.05 vs. SCI; c, *P* < 0.05 vs. SCI + FMT; d, *P* < 0.05 vs. SCI + hADSCs.

Double-immunofluorescence analysis further showed increased TNF-α- and IL-1β-positive areas within Iba1-positive regions after SCI, with treatment-associated reductions that paralleled the overall TNF-α and IL-1β immunoreactivity patterns described above. Both measures were lowest in the combined-treatment group (**Figures 4G–I**). These findings indicate that the reduction in inflammatory cytokine signals after treatment was also evident within Iba1-positive myeloid regions. Because Iba1 does not distinguish resident microglia from infiltrating macrophages, these results were interpreted as changes in myeloid-associated inflammatory activity rather than as microglia-specific effects.

ELISA and qPCR analyses of spinal cord tissue showed corresponding changes in inflammatory mediators, including reductions in TNF-α, IL-1β, IL-6, and IL-18 and an increase in IL-10 following combined treatment (**Supplementary Figure S3**).

### 3.4 Combined treatment was associated with greater local hADSC retention and reduced chronic fibrotic scar and inflammatory burden

To assess the local persistence of transplanted hADSCs, spinal cord sections collected at 7 d.p.i. were stained for the human-specific nuclear antigen HuNu. HuNu-positive cells were detected within and around the lesion site in both the SCI + hADSCs and SCI + FMT + hADSCs groups (**Figure 5A**). Quantitative analysis showed a significantly higher density of HuNu-positive cells in the SCI + FMT + hADSCs group than in the SCI + hADSCs group (P<0.05) (**Figure 5B**). These findings indicate that transplanted human-origin cells remained detectable at the lesion site at 7 d.p.i. and that the combined-treatment group showed greater local retention of HuNu-positive cells. At 35 d.p.i., H&E and Masson’s trichrome staining showed extensive tissue loss and collagen deposition in the SCI group (**Figures 5C, D**). The collagen-positive fibrotic area was 1.16 ± 0.09 mm² in the SCI group and was reduced to 0.43 ± 0.08 mm² and 0.29 ± 0.05 mm² following FMT or hADSCs monotherapy, respectively. The combined-treatment group showed the smallest fibrotic area among the SCI groups (0.16 ± 0.03 mm²; **Figure 5E**). GFAP/CD68 double immunofluorescence at 35 d.p.i. further showed reduced CD68-positive inflammatory burden in the combined-treatment group, with CD68 signal decreasing from 55.83 ± 4.92 in the SCI group to 7.44 ± 1.42 following combined treatment (**Figures 5F, G**).

**Figure 5:**
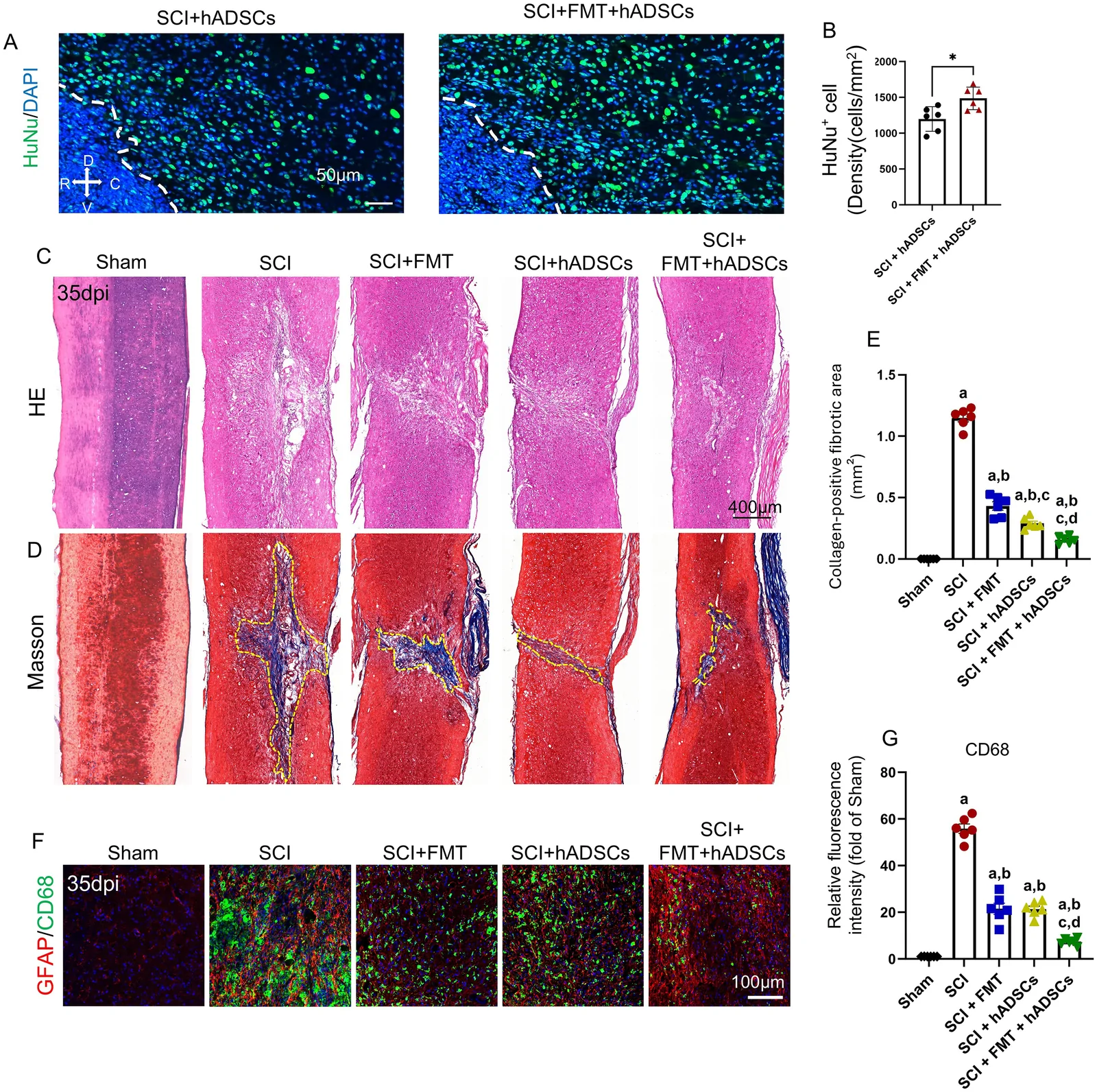
Local persistence of transplanted hADSCs and reduced chronic lesion and inflammatory burden after combined treatment. **(A)** Representative immunofluorescence images of the human-specific nuclear antigen HuNu (green) in spinal cord sections from the SCI + hADSCs and SCI + FMT + hADSCs groups at 7 d.p.i. Nuclei were counterstained with DAPI (blue). Dashed lines indicate the lesion boundary. Scale bar, 50 μm. **(B)** Quantification of HuNu-positive cell density within the lesion/perilesional region (n = 6 animals per group). **(C)** Representative H&E staining of spinal cord sections at 35 d.p.i. **(D)** Representative Masson’s trichrome staining at 35 d.p.i.; dashed lines outline the lesion/fibrotic region. **(E)** Quantification of collagen-positive fibrotic area in Masson-stained spinal cord sections. **(F)** Representative GFAP (red) and CD68 (green) double-immunofluorescence images at 35 d.p.i. Scale bar, 100 μm. **(G)** Quantification of relative CD68 fluorescence intensity, expressed as fold of Sham. For panels **(E, G)**, n = 6 animals per group. Data are presented as mean ± SD. For panel **(B)**, statistical comparison was performed using an unpaired two-tailed Student’s t-test. For panels **(E, G)**, one-way ANOVA followed by Bonferroni’s multiple-comparison test was used. a, P < 0.05 vs. Sham; b, P < 0.05 vs. SCI; c, P < 0.05 vs. SCI + FMT; d, P < 0.05 vs. SCI + hADSCs.

### 3.5 Combined FMT and hADSCs treatment enhanced chronic neural tissue preservation

NF200 immunofluorescence at 35 d.p.i. showed marked loss of neurofilament fibers in the SCI group (16.56 ± 5.83% of Sham; **Figures 6A, D**). FMT (42.10 ± 6.91%) and hADSCs (53.11 ± 6.27%) monotherapies partially preserved NF200 signal, whereas the combined-treatment group reached 79.29 ± 7.66%. NeuN staining followed a similar pattern, with the combined-treatment group exhibiting the greatest preservation of NeuN-positive neurons in the perilesional region (**Figures 6B, E**). LFB and Nissl staining further supported greater preservation of myelin and neuronal tissue following the combined treatment (**Figures 6C, F**).

**Figure 6:**
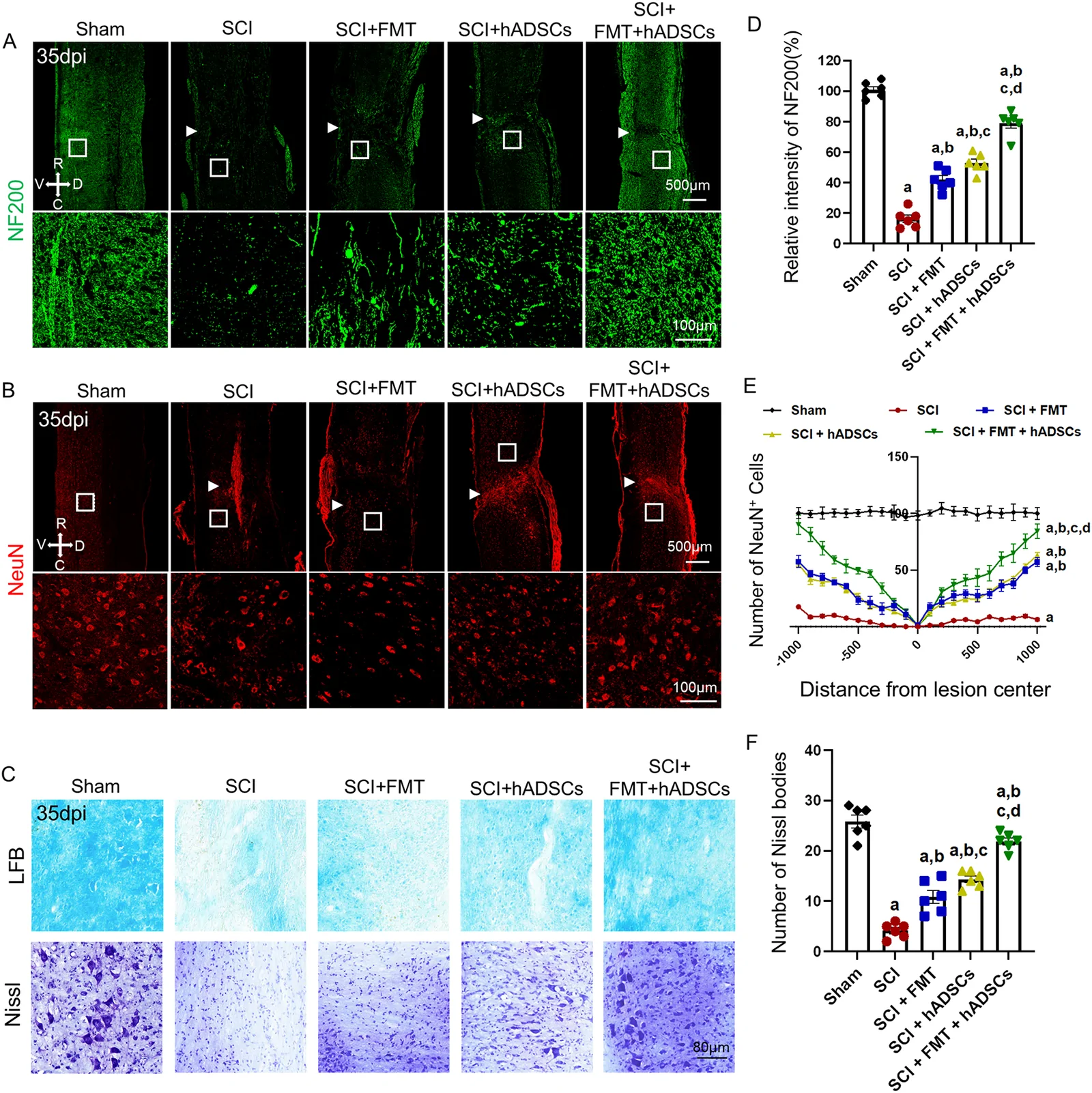
Combined FMT and hADSCs treatment enhanced chronic neural tissue preservation after SCI. **(A)** Representative NF200 immunofluorescence images at 35 d.p.i.; upper panels show low-magnification views and lower panels show higher-magnification images of the boxed regions. **(B)** Representative NeuN immunofluorescence images at 35 d.p.i.; upper panels show low-magnification views and lower panels show higher-magnification images of the boxed regions. **(C)** Representative Luxol fast blue (LFB) and Nissl staining at 35 d.p.i. **(D)** Quantification of NF200 fluorescence intensity normalized to the Sham group. **(E)** Spatial distribution of NeuN-positive cells according to distance from the lesion center. **(F)** Quantification of Nissl-positive neuronal profiles. n = 6 animals per group. Data are presented as mean ± SD. One-way ANOVA followed by Bonferroni’s multiple-comparison test was used for panels **(D, F)**; two-way ANOVA followed by Bonferroni-adjusted comparisons was used for panel **(E)** a, *P* < 0.05 vs. Sham; b, *P* < 0.05 vs. SCI; c, *P* < 0.05 vs. SCI + FMT; d, *P* < 0.05 vs. SCI + hADSCs.

### 3.6 Exploratory analysis identified treatment-associated differences in gut microbial composition after SCI

At 35 d.p.i., exploratory 16S rRNA sequencing was performed in the SCI, SCI + FMT, SCI + hADSCs, and SCI + FMT + hADSCs groups (n = 9 per group). Microbial richness differed significantly among the four groups, as assessed by the ACE (Kruskal–Wallis H = 22.26, P < 0.0001) and Chao1 (H = 21.24, P < 0.0001) indices. After Benjamini–Hochberg correction, the SCI group showed higher ACE and Chao1 values than the SCI + FMT and SCI + hADSCs groups, whereas the combined-treatment group showed higher richness than the SCI + hADSCs group but did not differ significantly from the SCI or SCI + FMT groups (**Supplementary Figure S4A**).

Bray–Curtis PCoA based on genus-level relative abundance profiles illustrated variation in overall microbial community structure among the four treatment groups. PERMANOVA detected a significant overall group effect (pseudo-F = 4.653, R² = 0.304, P = 0.0001). Five of the six pairwise comparisons remained significant after Benjamini–Hochberg correction, whereas the SCI + hADSCs versus SCI + FMT + hADSCs comparison did not reach statistical significance (adjusted P = 0.061). PERMDISP provided no evidence of significant differences in multivariate dispersion among groups (F = 0.627, P = 0.599), indicating that the overall PERMANOVA result was unlikely to be explained by unequal within-group dispersion (**Supplementary Figure S4B**). Complete pairwise PERMANOVA results are provided in the **Supplementary Source Data**.

Individual-sample stacked bar plots at the phylum and genus levels further illustrated variation in taxonomic composition within and among treatment groups (**Supplementary Figures S4C, D**). These profiles were presented descriptively without taxon-specific statistical or mechanistic interpretation. Because Sham, pre-injury baseline, and donor microbiota samples were unavailable, these findings were interpreted as exploratory treatment-associated differences among the four SCI groups rather than evidence of SCI-induced dysbiosis, restoration toward a healthy microbiota, or donor microbiota engraftment.

### 3.7 Fecal filtrate enhanced hADSCs paracrine secretion without altering cellular metabolic activity

To examine whether soluble components of donor fecal material could modulate hADSCs secretory activity, hADSCs were exposed to 5% (v/v) filtered donor fecal material for 24 h, washed three times with sterile PBS, and then cultured in fresh medium for 48 h before conditioned medium (CM) collection (**Figure 7A**). CCK-8 absorbance did not differ significantly between control and fecal-filtrate-preconditioned hADSCs, indicating no detectable change in cellular metabolic activity under these conditions (**Figure 7B**). ELISA of CM showed that fecal-filtrate-preconditioned hADSCs secreted significantly higher levels of VEGF (2.3-fold), HGF (2.2-fold), and IL-10 (1.8-fold) than untreated hADSCs (all *P* < 0.05; **Figures 7C–E**). BDNF and TSG-6 showed modest, non-significant increases (**Figures 7F, G**). IL-10 knockdown significantly reduced IL-10 secretion relative to fecal-filtrate-preconditioned hADSCs, whereas cellular metabolic activity and VEGF and HGF secretion were not significantly altered by IL-10 knockdown (**Figures 7B–E**). These findings indicate that exposure to filtered donor fecal material modulated hADSCs paracrine secretion *in vitro*, with increased release of VEGF, HGF, and IL-10.

**Figure 7:**
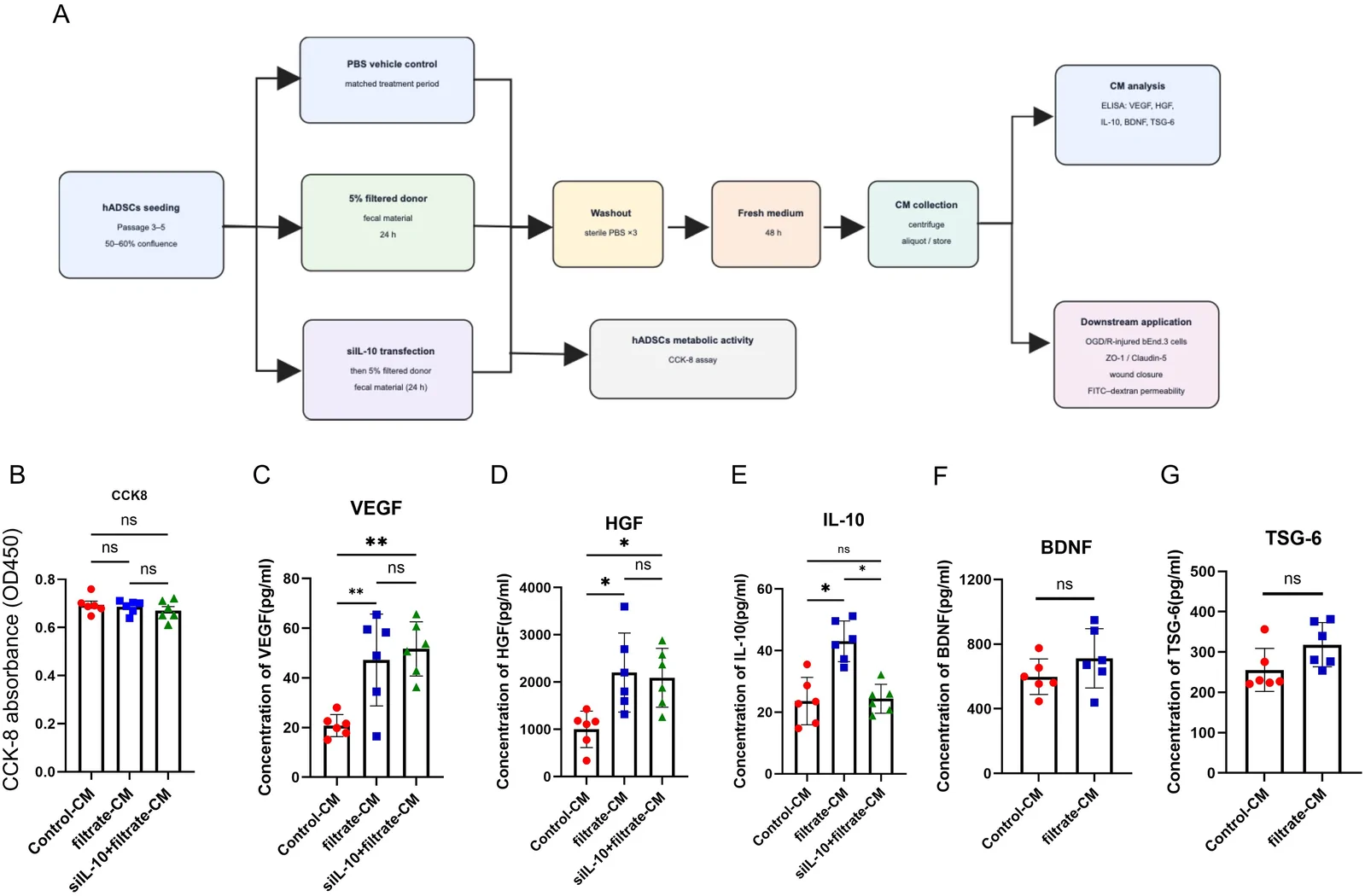
Fecal filtrate enhanced VEGF, HGF, and IL-10 secretion by hADSCs. Fecal-filtrate preconditioning increased VEGF, HGF, and IL-10 secretion by hADSCs. **(A)** Schematic of the *in vitro* experimental workflow, including hADSCs treatment, siIL-10 transfection, washout, conditioned medium (CM) collection, and downstream analyses. **(B)** CCK-8 assessment of hADSCs cellular metabolic activity following the indicated treatments. **(C–E)** ELISA quantification of VEGF **(C)**, HGF **(D)**, and IL-10 **(E)** concentrations in CM collected from control hADSCs, fecal-filtrate-preconditioned hADSCs, and IL-10-silenced hADSCs subsequently exposed to fecal filtrate. **(F, G)** ELISA quantification of BDNF **(F)** and TSG-6 **(G)** concentrations in CM from control and fecal-filtrate-preconditioned hADSCs. Data are presented as mean ± SD. n = 6 independent experiments. One-way ANOVA followed by Bonferroni’s multiple-comparison test was used for panels **(B–E)**, and an unpaired two-tailed Student’s *t*-test was used for panels **(F, G)** *P* < 0.05, P < 0.01, ns, not significant.

### 3.8 Fecal-filtrate-preconditioned hADSCs-CM enhanced endothelial barrier protection after OGD/R

To determine whether conditioned medium from fecal-filtrate-preconditioned hADSCs provided greater protection against OGD/R-induced endothelial injury, bEnd.3 cells were subjected to OGD/R and treated with conditioned medium from untreated or fecal-filtrate-preconditioned hADSCs. Wound closure at 12 h decreased from 80.56 ± 5.30% in control cells to 21.27 ± 1.52% after OGD/R (**Figures 2A, D**). Treatment with hADSCs-CM increased wound closure to 41.83 ± 3.94%, whereas fecal-filtrate-preconditioned hADSCs-CM further increased it to 59.04 ± 6.34% (*P* < 0.05 vs. hADSCs-CM; **Figures 2A, D**).

ZO-1 immunofluorescence showed a continuous junctional pattern under normoxic conditions that was markedly disrupted by OGD/R (**Figure 2B**). Relative ZO-1 fluorescence decreased from 1.01 ± 0.09 to 0.34 ± 0.04 after OGD/R, increased to 0.49 ± 0.02 with hADSCs-CM, and reached 0.64 ± 0.05 with fecal-filtrate-preconditioned hADSCs-CM (**Figure 2E**). Claudin-5 showed a similar pattern, decreasing from 1.07 ± 0.16 in control cells to 0.27 ± 0.05 after OGD/R and increasing to 0.52 ± 0.02 and 0.78 ± 0.04 following treatment with hADSCs-CM and fecal-filtrate-preconditioned hADSCs-CM, respectively (*P* < 0.05 vs. hADSCs-CM; **Figures 2C, F**).

Consistent with these junctional changes, FITC-dextran permeability across bEnd.3 monolayers increased after OGD/R and was reduced by hADSCs-CM, with a further reduction following treatment with fecal-filtrate-preconditioned hADSCs-CM (**Figure 2G**). IL-10 knockdown in fecal-filtrate-preconditioned hADSCs partially attenuated these effects, resulting in reduced wound closure, lower ZO-1 and Claudin-5 signals, and increased FITC-dextran permeability compared with fecal-filtrate-preconditioned hADSCs-CM (**Figures 2A–G**). These findings support a contribution of hADSCs-derived IL-10 to the endothelial-protective effects observed after fecal-filtrate preconditioning, while the incomplete reversal suggests that additional secreted factors may also contribute.

## 4 Discussion

The present study evaluated the therapeutic effects of combining FMT with hADSCs transplantation after SCI and examined whether soluble components of healthy-donor fecal material could modify hADSCs secretory activity *in vitro*. Three principal observations emerged. First, combined FMT and hADSCs treatment produced broader *in vivo* benefit than either monotherapy across multiple outcomes, including improved functional recovery, attenuation of BSCB disruption and inflammatory responses, and greater neural tissue preservation. Second, exposure to 0.22-μm-filtered fecal filtrate prepared from healthy donor mice increased hADSCs secretion of VEGF, HGF, and IL-10 *in vitro*. Third, conditioned medium from fecal-filtrate-preconditioned hADSCs provided greater protection to OGD/R-injured endothelial cells than conditioned medium from untreated hADSCs, and IL-10 knockdown partially attenuated these effects. Together, these findings support a therapeutic potential of combined FMT and hADSCs treatment while distinguishing the observed *in vivo* benefits from the reductionist *in vitro* evidence that hADSCs secretory activity can be modified by soluble fecal components.

Secondary injury after SCI is driven in part by reciprocal interactions between vascular dysfunction and inflammation (1, 2). BSCB disruption is an early event that permits plasma-derived molecules and inflammatory mediators to enter the injured cord, amplifying edema, immune activation, and neural tissue loss (2, 6). In the present study, reduced Evans blue extravasation together with greater ZO-1 and Claudin-5 coverage within CD31-positive vascular regions suggests that the combined treatment attenuated BSCB disruption more effectively than either monotherapy. These findings should be interpreted as evidence of BSCB preservation rather than complete restoration of barrier function. Nevertheless, limiting early barrier disruption may reduce the exposure of injured spinal tissue to blood-derived inflammatory mediators and thereby contribute to a less damaging secondary injury environment.

The inflammatory findings are consistent with this vascular interpretation. At 7 d.p.i., combined treatment reduced CD68, TNF-α, and IL-1β immunoreactivity, decreased tissue levels of TNF-α, IL-1β, IL-6, and IL-18, and increased IL-10. Double-immunofluorescence analysis further showed that TNF-α and IL-1β signals were increased within Iba1-positive myeloid regions after SCI and were reduced following treatment. Because neither CD68 nor Iba1 distinguishes resident microglia from infiltrating macrophages, these results are best interpreted as reduced myeloid-associated inflammatory activation rather than a lineage-specific effect (33). Barrier failure can amplify inflammatory infiltration and cytokine production, whereas inflammatory mediators can in turn aggravate endothelial dysfunction (2, 34). The present findings therefore support coordinated attenuation of the neurovascular–inflammatory response after combined treatment. However, the study design does not establish whether vascular preservation preceded inflammatory suppression or whether these processes developed in parallel.

The *in vitro* experiments were designed to determine whether hADSCs can respond to soluble components present in healthy-donor fecal material. Following exposure to healthy-donor fecal filtrate, VEGF, HGF, and IL-10 secretion levels were 2.3-, 2.2-, and 1.8-fold those of untreated hADSCs, respectively. The 0.22-μm filtration step was intended to remove bacterial cells and focus the experiment on soluble or filterable fecal components. However, the active constituents were not characterized and may include microbial metabolites, bacterial products, host-derived molecules, or other soluble factors. These experiments therefore demonstrate hADSC responsiveness to healthy-donor fecal soluble components *in vitro* but do not identify the molecular signals responsible for this response.

The pattern of factor induction is biologically relevant to vascular and inflammatory regulation. VEGF has been implicated in endothelial survival and vascular repair (35–37), whereas HGF promote endothelial migration and tissue repair (38–40). IL-10 has well-established anti-inflammatory activity and has also been implicated in the regulation of endothelial barrier integrity (41–43). Importantly, IL-10 silencing in fecal-filtrate-preconditioned hADSCs partially attenuated the protective effects of their conditioned medium on OGD/R-injured endothelial cells. This loss-of-function experiment supports a functional contribution of hADSC-derived IL-10 to the observed endothelial protection. However, the protective effect was not completely abolished, indicating that IL-10 is unlikely to be the sole mediator. VEGF and HGF were not subjected to direct loss-of-function testing and should therefore be regarded as candidate contributors rather than experimentally established mediators. In addition, the increase in IL-10 detected in spinal cord tissue *in vivo* cannot be attributed specifically to transplanted hADSCs because species-specific human IL-10 was not measured and host myeloid or other resident cells may also contribute.

The endothelial experiments further support this interpretation. Conditioned medium from fecal-filtrate-preconditioned hADSCs improved scratch-wound closure, preserved ZO-1 and Claudin-5 immunofluorescence signals, and reduced FITC-dextran permeability in OGD/R-injured bEnd.3 monolayers compared with conditioned medium from untreated hADSCs. Because hADSCs were seeded at comparable densities and conditioned media were collected under the same culture volume and incubation period, secreted factors were compared based on their concentrations in the conditioned medium. The absence of significant differences in CCK-8 absorbance among the relevant hADSC groups further suggested that the observed changes in VEGF, HGF, and IL-10 concentrations were unlikely to be explained solely by major differences in cellular metabolic activity. In addition, scratch-wound closure reflects the combined effects of cell migration and proliferation and should not be interpreted as a migration-specific endpoint. The bEnd.3 model also represents a simplified endothelial injury system rather than a complete *in vitro* reconstruction of the BSCB. Nevertheless, the combined junctional and permeability findings provide functional evidence that exposure of hADSCs to healthy-donor fecal filtrate can alter the endothelial-protective activity of their conditioned medium under the tested conditions.

The chronic-stage findings are compatible with this early vascular and inflammatory observations. Combined treatment was associated with reduced lesion and collagen-rich fibrotic burden, reduced chronic CD68-positive myeloid accumulation, and greater preservation of myelin, NeuN-positive neurons, and NF200-positive fibers. Early attenuation of BSCB disruption and inflammatory activation could plausibly contribute to a tissue environment that is more permissive to long-term structural preservation. However, the present data do not establish a direct temporal or causal sequence linking the acute BSCB findings to the chronic histological outcomes. The chronic findings should therefore be viewed as complementary evidence of broader tissue preservation rather than proof of a single barrier-centered mechanism.

The microbiota analysis provides exploratory information about treatment-associated community differences. ACE and Chao1 richness indices differed among the four SCI groups. The combined-treatment group showed higher ACE and Chao1 values than the SCI+hADSCs group but did not differ significantly from the SCI or SCI+FMT groups. Importantly, greater microbial richness should not necessarily be interpreted as a healthier microbial state. Bray-Curtis PCoA showed treatment-associated variation in overall community structure, and PERMANOVA identified a significant overall group effect, whereas PERMDISP did not indicate unequal within-group dispersion. However, the adjusted pairwise PERMANOVA comparison between the SCI+hADSCs and combined-treatment groups did not reach statistical significance. Phylum- and genus-level profiles were therefore presented descriptively without taxon-specific mechanistic interpretation.

Several limitations should be acknowledged. First, the *in vitro* fecal-filtrate model does not reproduce the systemic environment encountered by transplanted hADSCs after oral FMT. Circulating metabolites were not measured, serum or plasma from FMT-treated animals was not used for preconditioning, and transplanted hADSCs were not isolated for ex vivo analysis of their secretory state. The active components of the healthy-donor fecal filtrate were not characterized, and an SCI-derived fecal-filtrate control was unavailable. Therefore, the present study demonstrates that hADSCs can respond to soluble components present in healthy-donor fecal filtrate *in vitro* but does not establish that oral FMT directly reprograms transplanted hADSCs *in vivo*. In addition, although IL-10 knockdown supported a functional contribution of hADSC-derived IL-10 *in vitro*, VEGF and HGF were not subjected to loss-of-function validation and should be regarded as candidate mediators.

Second, the microbiota analysis was exploratory. Sham, pre-injury baseline, and donor microbiota samples were unavailable, and fecal samples were collected at a single endpoint. Accordingly, the data cannot establish SCI-induced dysbiosis relative to uninjured animals, restoration toward a healthy or baseline microbial state, or donor microbiota engraftment. Although all four SCI groups were processed in the same batch for DNA extraction, library preparation, and sequencing, this does not compensate for the absence of these controls. Moreover, all animals received the same perioperative antibiotic regimen, which minimized differential antibiotic exposure among groups but did not exclude a general influence of antibiotic administration on microbiota composition or FMT-associated changes. The 16S findings should therefore be interpreted as treatment-associated differences among the four SCI groups rather than evidence that microbiota remodeling mediated the therapeutic effects of the combined treatment.

Third, several model-related considerations remain. Human ADSCs were transplanted into immunocompetent mice, a commonly used preclinical approach that enhances translational relevance and is supported by the relatively low immunogenicity and immunomodulatory properties of mesenchymal stromal cells. HuNu staining confirmed short-term local persistence of transplanted human-origin cells at 7 d.p.i.; however, these findings do not establish long-term cell survival, viability, functional activity, systemic biodistribution, or complete immune tolerance. Species mismatch may therefore still have influenced cell persistence or host immune responses. In addition, bEnd.3 cells represent a simplified endothelial injury model and do not fully reproduce spinal cord microvascular endothelium or the multicellular architecture of the BSCB. The relatively small number of independent *in vitro* experiments further limits the strength of the mechanistic inference. The different treatment schedules reflected the distinct routes of administration of the two interventions: hADSCs were administered as a single intralesional injection to provide local cell-based treatment at the lesion site, whereas FMT was administered daily to provide repeated donor-derived intestinal exposure during the post-injury period. However, these schedules were neither directly compared nor optimized, and differences in dosing frequency and treatment duration may have influenced the magnitude and duration of the observed effects. Future studies using species-matched cell models, more detailed immune phenotyping, and more physiologically representative neurovascular systems will be required to address these issues.

Despite these limitations, the study provides evidence that combined FMT and hADSCs treatment produced greater therapeutic effects than either monotherapy across several functional, vascular, inflammatory, and histological outcomes. Because no formal statistical interaction analysis was designed to establish synergy, these effects should not be described as synergistic or as exceeding the sum of the individual treatments. The vitro findings further demonstrate that hADSC secretory activity can be modified by soluble components present in healthy-donor fecal filtrate and that the resulting conditioned medium has greater endothelial-protective activity under the tested conditions. These observations provide a rationale for future studies using circulating factors from FMT-treated animals, molecular characterization of the active fecal components, species-specific analysis of transplanted cell products, and more physiologically representative BSCB models to determine whether circulating factors generated after oral FMT exert comparable effects on transplanted hADSCs *in vivo*.

## 5 Conclusion

Combined FMT and hADSCs treatment was associated with greater BSCB preservation, reduced inflammatory responses, improved neural tissue preservation, and better functional outcomes after SCI than either monotherapy. *In vitro*, filtered healthy-donor fecal material increased hADSC secretion of VEGF, HGF, and IL-10, and conditioned medium from these preconditioned cells provided greater protection to OGD/R-injured endothelial cells. IL-10 knockdown partially attenuated this protection, supporting a contributory role for hADSC-derived IL-10 in the *in vitro* response. However, the present findings do not establish that oral FMT directly reprograms transplanted hADSCs *in vivo* or that treatment-associated microbiota changes mediate the therapeutic effects of the combined intervention. Further work is required to identify the relevant circulating or fecal-derived signals and determine whether this interaction occurs within the injured spinal cord.

## Data Availability

The raw 16S rRNA gene amplicon sequencing data generated in this study have been deposited in the NCBI Sequence Read Archive under BioProject accession number PRJNA1518846. Processed microbiome data, including taxonomic abundance tables, alpha-diversity data, and beta-diversity statistical outputs, are provided in the **Supplementary Materials**. Additional source data supporting the quantitative analyses are available in the accompanying **Supplementary Source Data**.
