# Supplementary material for: Combined fecal microbiota transplantation and adipose-derived stem cells protect the blood-spinal cord barrier after spinal cord injury
Source: Front Immunol. 2026 Oct 13;17:1877863. doi: 10.3389/fimmu.2026.1877863 (PMC13709993; doi:10.3389/fimmu.2026.1877863)
Supplement: Supplementary file 1 [file SupplementaryFile1.docx]

Supplementary Material

# 1 Supplementary Data

# 2 Supplementary Figures and Tables

## 2.1 Table

| **No.** | **Antibody** | **Working Dilution** | **Manufacturer** | **Catalog Number** |
| --- | --- | --- | --- | --- |
| 1 | anti-NeuN | 1:200 | Sigma | MAB377 |
| 2 | anti-NF200 | 1:400 | Abcam | ab8135 |
| 3 | anti-IL-1β | 1:200 | ABclonal | A22257 |
| 4 | anti-TNF-α | 1:100 | Santa Cruz | sc-52746 |
| 5 | anti-CD68 | 1:200 | Bio-Rad | MCA1957GA |
| 6 | anti-CD31/PECAM-1 | 1:300 | BD Biosciences | 550274 |
| 7 | anti-ZO-1 | 1:200 | ABclonal | A25306 |
| 8 | anti-Claudin-5 | 1:200 | ABclonal | A25830 |
| 9 | anti-GFAP | 1:800 | Sigma | mab360 |
| 10 | anti-Iba-1 | 1:200/1:500 | Abcam/ Wako | ab283319/ 019–19741 |
| 11 | anti-HuNu | 1:100 | Millipore | MAB1281 |

**Table1. Primary antibodies for immunofluorescence, with applied dilutions**

| Genes | Forward primer sequence (5′– 3′) | Reverse primer sequence (5′– 3′) |
| --- | --- | --- |
| IL-18 | 5′-ATGCCTGATATCGACCGAAC-3′ | 5′-TGGCACACGTTTCTGAAAGA-3′ |
| IL-1β | 5′-CAGCAGCATCTCGACAAGAG-3′ | 5′-CATCATCCCACGAGTCACAG-3′ |
| TNF-α | 5′-TACTGAACTTCGGGGTGATCG-3′ | 5′-CCACTTGGTGGTTTGCTACG-3′ |
| IL-6 | 5′-CTTCCATCCAGTTGCCTTCTTG-3′ | 5′-AATTAAGCCTCCGACTTGTGAAG-3′ |
| IL-10 | 5′-CACTCCCTGCATTACAATC-3′ | 5′-CAATGATGGTATTATAGGATCCC-3′ |
| GAPDH | 5′-GAACATCATCCCTGCATCCA-3′ | 5′-CCAGTGAGCTTCCCGTTCA-3′ |

**Table 2. Sequences of primer pairs**

## 2.2 Supplementary Figures

**Supplementary Figure S1. Animal enrollment, mortality, and allocation to experimental endpoints.**
A total of 132 mice entered the study. Nineteen animals died before the scheduled experimental endpoints, leaving 113 unique animals included in at least one analysis. Animals were allocated to Evans blue/NIRF assessment at 7 d.p.i., acute immunofluorescence at 7 d.p.i., chronic histology/immunofluorescence at 35 d.p.i., and 16S rRNA gene sequencing at 35 d.p.i., as indicated. Behavioral assessments were performed longitudinally in an overlapping subset of animals subsequently used for 35 d.p.i. endpoint analyses and were therefore not counted as a separate cohort. Final sample sizes for each analysis are shown in the flow diagram.

**Supplementary Figure S2.** Characterization of hADSCs used for transplantation. **(A)** Spindle-shaped morphology of passage-3 primary hADSCs under phase-contrast microscopy. Trilineage differentiation of hADSCs demonstrated by Alizarin Red S staining for osteogenesis **(B)**, Oil Red O staining for adipogenesis **(C)**, and Alcian Blue staining for chondrogenesis **(D)**. **(E)** Flow cytometric analysis showing the mesenchymal immunophenotype of hADSCs.

**Supplementary Figure S3. Molecular assessment of acute inflammatory responses after SCI.**
**(A)** qPCR analysis of TNF-α, IL-1β, IL-6, IL-18, and IL-10 expression in spinal cord tissue at 7 d.p.i. **(B)** ELISA quantification of TNF-α, IL-1β, IL-6, IL-18, and IL-10 in spinal cord tissue at 7 d.p.i. Data are presented as mean ± SD. n = 6 animals per group. Statistical comparisons were performed using one-way ANOVA followed by Bonferroni’s multiple-comparison test. ^a^P < 0.05 vs. Sham; ^b^P < 0.05 vs. SCI; ^c^P < 0.05 vs. SCI + FMT; ^d^P < 0.05 vs. SCI + hADSCs.

**Supplementary Figure S4. Exploratory gut microbiota profiling after SCI.**
**(A)** ACE and Chao1 richness indices among the SCI, SCI + FMT, SCI + hADSCs, and SCI + FMT + hADSCs groups (n = 9 per group). Group differences were assessed using the Kruskal–Wallis test followed by Dunn’s pairwise comparisons with Benjamini–Hochberg correction. **(B)** Bray–Curtis principal coordinates analysis (PCoA) of genus-level relative abundance profiles. Overall group differences were assessed by PERMANOVA, and homogeneity of multivariate dispersion was evaluated by PERMDISP. **(C, D)** Individual-sample relative abundance profiles at the phylum **(C)** and genus **(D)** levels. Taxonomic profiles are shown descriptively without taxon-specific statistical interpretation.
